# Long-term visual acuity results from cataract surgery and its association with self-reported visual function: Catquest applicability

**DOI:** 10.5935/0004-2749.2022-0198

**Published:** 2022-10-19

**Authors:** Andre Hiroshi Bando, Koiti Uchida Hamada, Vinícius Pereira Leite Nakamura, Ricardo Y. Abe, Pedro Vanalle Ferrari, Bruno Torres Herrerias, Flavio Eduardo Hirai, Carolina P. B. Gracitelli

**Affiliations:** 1 Glaucoma Service, Department of Ophthalmology and Visual Science, Escola Paulista de Medicina, Universidade Federal de São Paulo, São Paulo, SP, Brazil; 2 Hospital Oftalmológico de Brasília, Brasília, DF, Brazil; 3 Centro de Estudos Alcides Hirai, VER MAIS Oftalmologia, Vinhedo, SP, Brazil

**Keywords:** Cataract extraction, Visual acuity, Surveys and questionnaires, Quality of life, Patient reported outcome measures, Extração de catarata, Acuidade visual, Inquéritos e questionários, Qualidade de vida, Medidas de resultados relatados pelo paciente

## Abstract

**Purposes:**

This study aimed to determine the association of the long-term refractive outcomes of cataract surgery with self-reported visual function obtained using Catquest-9SF.

**Methods:**

Patients recruited from the cataract outpatient clinic of *VER MAIS Oftalmologia* underwent a complete ophthalmologic examination. Patients who were diagnosed with cataract with indications for phacoemulsification and intraocular lens implantation received the Catquest-9SF questionnaire before and after surgery at 30 days and 1 year.

**Results:**

A total of 133 patients were recruited, but 32 patients were lost to follow-up; finally, data from 101 patients (48 men, 53 women) were analyzed. The crude variance explained by the data was 69.9%, and the unexplained variance in the first contrast was 2.39 eigenvalues (>2); thus, these results are different from those expected from random data. The people separation index was 2.95 (>2), and the people trust value was 0.9 (>0.8). These indices were evaluated in the assessment of skill levels. Visual acuity was the main variable that correlated with the Catquest score.

**Conclusions:**

The Catquest-9SF translated into Portuguese proved to be a one-dimensional and psychometrically valid tool to assess visual dysfunction in patients with cataract, and it is successful in objectively quantifying improvements after surgery. The results of this tool could be predictive and concordant of visual acuity improvement.

## Abstract (pt)

**Objetivo:**

Associar os resultados refrativos a longo prazo da cirurgia de catarata e a função visual autorreferida pelo questionário Catquest-9SF.

**Métodos:**

Paciente recrutados no ambulatório de catarata da VER MAIS Oftalmologia, foram submetidos a exame oftalmológico completo. Após diagnóstico de catarata com indicação de tratamento cirúrgico com facoemulsificação e implante de lente intraocular, o questionário foi aplicado antes da intervenção, 30 dias após cirurgia e 1 ano após, novamente.

**Resultados:**

Foram recrutados 133 pacientes. No decorrer do seguimento, 32 pacientes foram perdidos e ao final foram analisados os dados de 101 pacientes, dos quais 48 foram homens e 53 foram mulheres. A variância bruta explicada por dados foi de 69,9% e a inexplicada em primeiro contraste por 2,39 eigenvalores, sendo maior que 2, o que nos mostra que são resultados differentes dos esperados de dados aleatórios. O índice de separação de pessoas foi de 2.95 (>2) e o valor de confiança de pessoas foi de 0,9 (>0,8). Estes índices são os valores mínimos aceitáveis na diferenciação de níveis de habilidade. Acuidade visual foi a principal variável correlacionada com o *score* do Catquest.

**Conclusões:**

O Catquest-9SF traduzido para o português se demonstrou unidimensional e uma ferramenta psicometricamente válida para avaliar disfunção visual em pacientes com catarata, além de ter tido sucesso para quantificar objetivamente melhoras após a intervenção cirúrgica. Essa ferramenta pode ser utilizada como preditiva e concordante da melhora da acuidade visual.

## INTRODUCTION

Cataract is the leading cause of visual impairment (VI) and reversible blindness worldwide^(1)^. A recent systematic review pointed that cataract, an important cause of VI, is a problem for a large and increasing number of people globally. In 2020, approximately 1.1 billion people were living with any causes of VI, and this is projected to increase to 1.8 billion in 2050^(2)^. Cataract surgery is an economically advantageous procedure that significantly improves the visual acuity and quality of life of these patients^(3)^. Given the recent advances in new intraocular lenses (*e.g.,* multifocal lens) and surgical technologies (e.g., femtosecond laser-assisted cataract surgery), better refractional results and more predictable quality of vision are expected^(3)^.

However, to determine whether a new intervention is worth the risk and cost, there is a need to find objective and reliable indicators for both preoperative and postoperative visual status^(4)^. In addition, an accurate preoperative assessment becomes important for both surgical decision-making and realistic expectations of the patient about the treatment^(5)^. Several functional questionnaires related to vision and its effect on patients’ quality of life were developed, such as the NEI-Visual Functioning Questionnaire 25^(6)^, Visual Function-14^(7)^, and Daily Vision Activities Scale for Cataract Surgery^(7,8)^. However, these questionnaires showed limited targeting in some populations, making other additional items necessary to facilitate measurement.

More recently, the Catquest questionnaire was developed to assess difficulties perceived by patients in their daily lives because of cataract^(9,10)^. In 2009, a new version of the questionnaire, i.e., Catquest-9SF, was validated and used in other populations, showing the same efficacy and results as the first version^(9)^. Recently, the same questionnaire has already been validated in other populations (Spanish, Chinese, and Australian)^(11)^ and was applied to diseases other than cataract, such as after corneal transplant surgery^(12)^. Last year, Antunes et al. validated the Portuguese version of Catquest-9SF in Minas Gerais, Brazil. In this context, this study aimed to validate the Catquest-9SF questionnaire into a different Brazilian population and find its association with long-term refractive outcomes.

## METHODS

This longitudinal prospective study was approved by the Institutional Review Board (IRB) of the Federal University of São Paulo (IRB number 1528.0055.12/2017). Written informed consent was obtained prospectively from all the participants, and all study procedures adhered to the tenets of the Declaration of Helsinki.

### Study participants

A total of 133 patients with cataract were included. All patients underwent a comprehensive ophthalmologic examination, which included review of medical history, visual acuity, slit-lamp biomicroscopy, intraocular pressure measurement (using the Goldmann tonometer), gonioscopy, and dilated fundoscopic examination. In addition, all patients answered the revised Catquest questionnaire three times (before surgery, 1 month after surgery, and 1 year after surgery). All patients provided sociodemographic information including age, sex, educational level, race, comorbidities, medications, and eye drops used.

The individuals with cataract included in the study were those aged >18 years who presented to the Cataract Division of the *VER MAIS Oftalmologia* Clinic for cataract surgery and had lens opacity of C1 (cortical opacity), NC1 (nuclear stain), or NO1 (nuclear opacity), according to the LOCS III classification system^(13)^.

Patients were excluded if they had or were diagnosed with glaucoma or ocular hypertension during the study, were using other eye drops or medications that could possibly alter the corneal surface, had self-referred diagnosis of depression or other psychiatric changes that make the assessment of the quality of life through self-report questionnaires difficult, or had any previous eye surgeries.

### Catquest-9SF questionnaire

The Catquest-9SF questionnaire consists of seven items regarding performance of daily living activities and two global items on general difficulties and satisfaction with vision. For the levels of perceived difficulty, the responses were as follows: 1, very high difficulty; 2, great difficulty; 3, some difficulty; and 4, no difficulty. For vision satisfaction, the responses were as follows: 1, very dissatisfied; 2, very dissatisfied; 3, quite satisfied; and 4, very satisfied. Lower scores generally indicate worse visual function. Details of the validation has been published elsewhere^(14)^.

Patients’ expectations regarding the outcomes of cataract surgery were assessed based on the results of the questionnaire survey conducted before surgery. Patients who reported any difficulty in Catquest-9SF were asked to estimate the improvement they expected: 1, a little; 2, a moderate amount; 3, a great deal; or 4, do not know. Based on these responses, we calculated an expected postoperative Catquest-9SF score for each patient by adjusting each preoperative item-specific score upward by the amount of expected postoperative improvement as stated previously. If an expected score exceeded 4, it was recorded as a 4. The summary score was calculated for seven items. The psychometric properties of the Catquest-9SF were evaluated with Rasch analysis using WINSTEPS (version 3.92.1)^(15)^.

### Demographic and socioeconomic parameters

Socioeconomic and clinical parameters were also evaluated to take into account potential confounding factors. All participants completed a questionnaire to obtain information regarding sex, ethnicity, and educational level (high school or no high school). The presence of systemic diseases was also determined by examination of medical records, medications, and participant recall. Specifically, comorbidities including systemic hypertension, diabetes mellitus, arthritis, heart disease, stroke, depression, cancers, and asthma were recorded. The comorbidity index was calculated by the sum of some scores given to each item.

### Statistical analysis

In the descriptive analysis, values with a normal distribution are presented as the mean and standard deviation, whereas those that were not distributed normally were presented as the median. The skewness-kurtosis test was used to confirm whether the distribution was normal or not. The *t*-test was used for multiple comparisons between pre- and postoperative measurements, and for variables without normal distribution, the corresponding nonparametric test (Wilcoxon rank test) was performed. Percentages were used to describe catego rical values and achieve better comparators between the two groups. All statistical analyses were performed using Stata version 13 (StataCorp LP, College Station, TX). The alpha level (type I error) was set at 0.05.

## RESULTS

Initially, there were 133 participants in the study; however, during the study period, 32 participants were lost during follow-up because of causes unrelated to the study. Finally, a total of 101 patients were enrolled, of which 48 were men and 53 were women. The mean age was 65.94 ± 13.08 years, 59.84% were Caucasians, and 53.38% were men. Regarding baseline visual acuity, the mean was 0.84 ± 0.81 LogMAR. Table 1 summarizes the demographic characteristics of the entire group at baseline and after 3 months follow-up.

**Table 1:** Baseline demographic findings of the entire group

Entire group (n=38)	Baseline	3 months follow-up	p-value
Age ± SD (years)	65.94 ± 13.08	NA	NA
Sex, male (%)	71 (53.38%)	NA	NA
Race, Caucasian (%)	76 (59.84%)	NA	NA
Visual acuity (LogMAR)	0.84 ± 0.81	0.25 ± 0.54	**p<0.001**
Spherical equivalent (Diopter)	-0.25 ± 2.31	-0.29 ± 0.64	**p=0.825**

**Figure 1:**
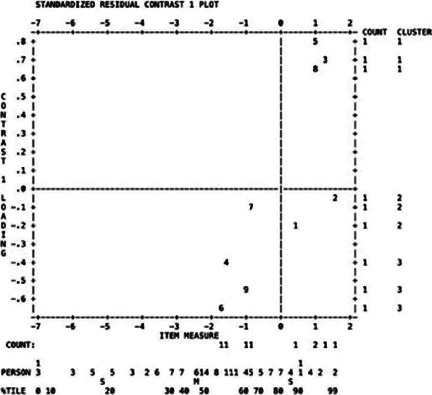
Unidimensionality analysis using standardized residual plot from the first contrast of the Catquest-9SF.

Our analysis revealed that Q2 was misfitted (with infit and outfit values of MNSQ of 1.47 and 2.13, respectively). We evaluated the principal components analysis of the residuals (difference between the observed and expected responses) to investigate unidimensionality^(15)^. Data were considered unidimensional if most of the variance is explained by the principal component and there is no significant explanation of the residual variance by the contrasts to the principal component^(15)^. The raw variance explained by measures was 69.9%, and the unexplained variance in the first contrast was 2.39 eigenvalue units (>2.0), which shows that these results are different from those expected from random data. The person separation index (PSI) is the ratio of the variance in person measures for the sample to the average error in estimating these measures, and the person reliability index (PRI) is the probability that a higher test performer has a corresponding higher ability in real life, whereas a lower performer has a lower ability. We found a PSI of 2.95 and a PTV of 0.90 (Figure 1), which were well above PSI >2 and PRI >0.8, the minimum for a good separation in skill levels.

**Figure 2:**
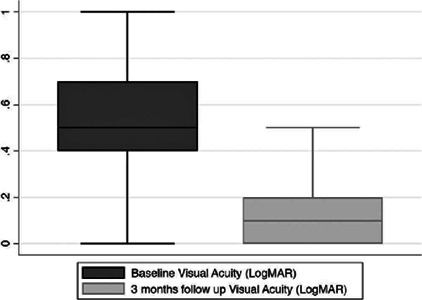
Boxplots showing the distribution of visual acuity (LogMAR) at baseline and after 3 months of follow-up. Box represents the median and interquartile range. Whiskers correspond to the maximum and minimum 1.5 IQR.

The best-corrected visual acuity in LogMAR with the best optical correction showed a significant correlation with the Catquest-9SF logit score. A significant positive correlation was found between the two measurements (r=0.282 and p=0.004), that is, the variable visual acuity (LogMAR) increases while the questionnaire score increases. Therefore, the greater the VI, the higher the Catquest scale score. Figure 2 illustrates the visual acuity improvement after cataract surgery.

In the long-term follow-up (3 months after surgery), no complications regarding cataract surgery occurred, and no patients underwent capsulotomy.

## DISCUSSION

In this study, the Rasch analysis showed that the Portuguese-translated Catquest-9SF is a reliable and valid psychometric tool for the assessment of visual function in patients with cataracts. The original Catquest-9SF questionnaire is known to evaluate visual function on patients with cataract for years^(16,17)^ and have been validated to other languages as well^(18,19)^.

Lundström et al., in an 11-year longitudinal study of 42.023 eyes that had undergone cataract surgery, used the Catquest-9SF questionnaire before and after surgery, which demonstrated stability at reliability, precision, and targeting^(18)^. In the same study, the mean postoperative visual acuity improved over 11 years according to the results of the questionnaire survey (0.14-0.09 logMAR, p<0.001).

Adnan et al. validated Catquest-9SF in Malay and Chinese^(19)^. Their results agree with our results, as both showed that this questionnaire is easy, understandable, and cost- and time-effective to administer, encouraging a high participant response rate, as shown by PSI of 2.84 for Malay and 2.59 for Chinese and PRI of 0.87 and 0.84, respectively. However, one limitation of the study by Adnan et al. is that the cohort only comprised patients with cataract preoperatively; thus, the responsiveness of the questionnaires (*i.e.,* change in visual disability) after the cataract surgery was not tested. In the present study, we showed improvements in the visual acuity and a positive correlation between visual acuity and Catquest-9SF scores.

The original Catquest, in which the Catquest-9SF was derived, was designed based on the classical test theory (CTT), the most common statistical tool to design and assess questionnaires. It is a psychometric paradigm that uses summary scoring of its variables^(16)^. Despite its popularity, it is not flawless: there is not an explicit ordered continuum along a unidimensional construct; therefore, there are no predetermined upscaling steps among data, given the unequal sizes between data^(16)^. To exemplify, the CTT would suggest that a questionnaire response of “very great difficulties” translated in numbers as 4 is assumed to be exactly two times greater than a score of 2 for “some difficulties,” but this is neither an accurate representation of the reality nor they are values arithmetically correlated among each other. Thus, a disadvantage of using CTT is the disparity between responses given on the questionnaires and actual clinical outcomes. Recently, to obtain more trustworthy questionnaire results, the item response theory is used, which is similar to the Rasch analysis^(20)^.

This study has some limitations. This study analyzed a limited number of eyes, and patients were lost through years of follow-up. Even though the minimum number for analysis is reduced by the Rasch method, our sample was still smaller than those in previous studies, which could lead to a loss of representativeness and reproducibility. However, even with a smaller sample, we managed to validate a questionnaire with good precision, reliability, and targeting of each question. Throughout the research period, some patients were lost to follow-up because some of our patients were very old and the pandemic represented a great difficulty to continue proper follow-up.

The Portuguese version of the Catquest-9SF was successfully validated using the Rasch analysis. We managed to compare the questionnaire results before and after the surgery, contributing to the existing evidence that the Catquest-9SF is a potential clinical tool to assess long-term visual function and patient’s satisfaction with surgery. The results of the questionnaire are predictive and concordant with visual acuity improvements.
